# Synthesis and Characterization of Novel Copper Nanoparticles for the Control of Leaf Spot and Anthracnose Diseases of Olive

**DOI:** 10.3390/nano11071667

**Published:** 2021-06-24

**Authors:** Panagiota Ntasiou, Alexandra Kaldeli Kerou, Theodora Karamanidou, Afrodite Vlachou, George T. Tziros, Alexander Tsouknidas, George S. Karaoglanidis

**Affiliations:** 1 Laboratory of Plant Pathology, Faculty of Agriculture, School of Agriculture, Forestry and Natural Environment, Aristotle University of Thessaloniki, P.O. Box 269, 54124 Thessaloniki, Greece; pntasiou@windowslive.com (P.N.); gtziros@yahoo.gr (G.T.T.); 2 PLiN Nanotechnology S.A., Spectra Business Center 12th km Thessaloniki-Chalkidiki, Thermi, 57001 Thessaloniki, Greece; ak@plin-nanotechnology.com (A.K.K.); tk@plin-nanotechnology.com (T.K.); av@plin-nanotechnology.com (A.V.); 3 Laboratory for Biomaterials and Computational Mechanics, Department of Mechanical Engineering, University of Western Macedonia, Bakola & Sialvera, 50132 Kozani, Greece

**Keywords:** antifungal activity, copper nanoparticles, CuNPs, nanofungicides, olive anthracnose, olive leaf spot

## Abstract

Olive crop is frequently treated with copper fungicides to combat foliar and fruit diseases such as olive leaf spot caused by *Fusicladium oleagineum* and anthracnose caused by *Colletotrichum* spp. The replacement of copper-based products with more eco-friendly alternatives is a priority. Metal nanoparticles synthesized in several ways have recently revolutionized crop protection with applications against important crop pathogens. In this study, we present the development of four copper-based nanoparticles (CuNP Type 1 to 4) synthesized with a wet chemistry approach. The CuNPs were characterized using Transmission Electron Microscopy, Dynamic Light Scattering, Laser Doppler Electrophoresis, and Attenuated Total Reflection measurements. In addition, the activity of the four CuNP types was tested in vitro and in planta against *F. oleagineum* and *Colletotrichum* spp. In vitro sensitivity measurements showed that for both pathogens, mycelial growth was the most susceptible developmental stage to the tested compounds. Against both pathogens, CuNP Type 1 and Type 2 were found to be more active in reducing mycelial growth compared to the reference commercial compounds of copper oxide and copper hydroxide. In planta experiments showed that CuNP Type 3 and CuNP Type 4 exhibited a strong protectant activity against both *F. oleagineum* and *Colletotrichum acutatum* with control efficacy values significantly higher than those achieved by the applications of either reference product.

## 1. Introduction

During the last two decades, engineered nanomaterials have attracted great experimental and research interest for applications in agriculture that include the delivery of genetic material, nutrients, and pesticides, the stabilization of biopesticides, and the development of nanobiosensors for pathogen detection or pesticide sensing [1,2]. Until now, several metal (silver, zinc, copper, magnesium, titanium) nanoparticles have been synthesized and evaluated for their antimicrobial activity against fungal and bacterial plant pathogens [3,4,5], while in other cases, nanoparticles have been utilized as carriers of conventional organic fungicides [6]. Most of the developed nanoproducts can be an excellent tool to reduce pesticide input into the environment by application at low dosages, since they ensured efficacy against the tested targets equal or higher to than that of commercially available conventional pesticides.

Copper fungicides have been used against several fungal and bacterial plant pathogens for more than 100 years [7]. Currently, several copper compounds (i.e., copper sulfate, copper carbonate, copper hydroxide, copper oxide, copper oxychloride, etc.) are used both in organic and conventional horticultural production. However, despite their worldwide large-scale use, copper fungicides may be harmful for non-target species and the environment. Recent advancements in nanotechnology may provide solutions to overcome limitations in excessive copper fungicides use, mostly through the reduction of application doses. Among the various metal nanoparticles that developed and were evaluated for their antimicrobial activity, copper nanoparticles (CuNPs) became the major weapon because of the lower production cost and their ubiquitous availability [8]. Several recent studies have shown that CuNPs, synthesized in several ways, have a great potential to combat a wide range of plant pathogenic fungi and oomycetes causing diseases on several crops [9,10,11,12]. The very small size of nanoparticles allows a more efficient penetration of the active ingredient into the microbial cell that in turn allows a more effective control with lower application doses [13]. Despite the fact that several studies cited earlier provided evidence on the antimicrobial activity of CuNPs against several phytopathogens, the limited is the information available on the mechanism corresponding to their antimicrobial action. The generation of Reactive Oxygen Species (ROS) after the absorption of Cu ions by the microbial cell wall has been proposed as the main mechanism of CuNPs antimicrobial activity [8,11,14].

Olive leaf spot (also known as peacock eye spot or olive scab) disease caused by *Fusicladium oleagineum* (syn. *Spilocaea oleagina* or *Cycloconium oleagineum*) is the main foliar disease of olive wherever in the world it is cultivated [15]. The pathogen infects mostly the leaves where circular, dark green oily spots appear on the adaxial leaf surface surrounded by a yellow chlorotic halo. The spots in the advanced stages of infection turn to dark brown, because of the presence of conidia and the increase in number and size covering the leaf surface. Lesions can also occur on petioles, fruit, and fruit peduncles, but such symptoms are relatively rare. Heavy infections cause defoliation and reduce oil yield and quality, while infections on the table-olive fruit cause blemishes on the fruit surface [16]. Infections by the pathogen occur in olive groves through autumn to late spring, while during the dry and hot summer conditions prevailing around Mediterranean countries, the pathogen remains dormant [15,17]. Infected leaves that remain on the trees during the summer are the main source of inoculum for autumn infections [15]. Rain-splashed conidia are dispersed on the tree canopy, causing infections throughout early autumn to early summer under wet and mild temperature conditions [17].

Olive anthracnose caused by *Colletotrichum* spp. is the most important disease of olive fruit occurring throughout the world. Currently, 13 different *Colletotrichum* species are associated to anthracnose of olive, among which six species belong to the *C. acutatum* species complex and two species belong to the *C. gloeosporioides* species complex [18,19]. In Greece, although the disease is known since 1920, its precise etiology only recently was elucidated, suggesting that the *C. acutatum* species complex is the predominant disease agent [20]. The most destructive disease symptoms occur most often on mature fruit in the form of brown-dark sunken rotten lesions that under moist conditions are covered by orange conidium masses [21]. Infected fruit are mummified and either drop onto the ground or remain attached on the trees, providing inoculum for primary latent infections that will occur during the spring on olive inflorescences [21,22]. In addition to mature fruit rot, leaf wilting and branch dieback may appear as a consequence of toxins production by the pathogen [23]. The disease, in addition to quantitative reduction in the yield, deteriorates heavily the olive oil quality by affecting its physicochemical, organoleptic, and sensory characteristics [19,24].

The control of both olive leaf spot and anthracnose is based on the integration of several measures that include sanitation practices, the use of disease-tolerant cultivars, and the application of fungicides. Among the chemical fungicides, copper compounds play a dominant role for the control of both diseases worldwide, since applications of target-site fungicides are restricted by the fact that most of them are soluble in the fruit oil [25]. Conventional copper formulations such as copper hydroxide or copper oxide are registered for use against olive fungal and bacterial diseases throughout the world. They have a broad activity spectrum that includes the major fungal pathogens of olive (*Colletrotrichum* spp., *F. oleagineum*, and *Pseudocercospora cladosporioides*) and the bacterial agent of olive knot disease, *Pseudomonas savastanoi*. In addition, they show low toxicity to olive trees, have long persistence on the trees, have low cost relative to that of target-site inhibitors, and have a low risk for fungicide resistance development, while their use is compatible with both organic and conventional farming [25,26]. On the other hand, copper fungicides are associated with several environmental risks and toxicity problems because of the associated copper residues in fruit, oil, and in addition, to the soil of olive orchards [7,27]. Copper compounds exhibit low mobility in the soil, and they tend to accumulate and persist in the soils of the olive groves for a long time [7,28].

Taking into account that olive crop is heavily treated with copper-based fungicides, Cu-minimizing measures are a priority to reduce the risk for environmental damages imposed by Cu accumulation in the Mediterranean environment. Such measures may include the use of plant extracts [29], microbial antagonists [30], systemic acquired resistance inducers [31,32], or novel formulations of conventional fungicide products such as copper-based products [25]. The current study was conducted aiming to (a) present the development of four novel Cu-based NPs—we provide providing data related to their physicochemical characterization—and (b) determine the antifungal activity of these products in vitro and in vivo against two major olive pathogens *F. oleagineum* and *Colletotrichum* spp.

## 2. Materials and Methods

### 2.1. Synthesis of Copper-Based Nanoparticles

Four types of Cu-based NPs were synthesized with a modified wet chemistry approach [5]. An overview of the process is being provided here, so as to ease the interpretation of the results and exhibit variations to the tested Cu types initially presented [5].

Two types of copper precursor salts were employed: (a) Copper (II) nitrate hemi-pentahydrate (>98% Cu(ΝO_3_)_2_·2.5H_2_O, Mr = 232.59 g/mol, Alfa Aesar, Haverhill, MA, USA) was used for Cu-NPs Types 1–3, and (b) Copper (II) chloride Dihydrate (99^+^% CuCl_2_·2H_2_O, Mr = 170.48 g/mol, CHEM-LAB, Zedelgem, BE) was used for Cu-NPs Type 4, respectively. Sodium hydroxide (>98% NaOH, CHEM-LAB, Zedelgem, BE) was used in all cases as the coordination- and pH-adjusting agent.

Three stabilizers were tested: (a) an animal protein (20,000–25,000 g/mol and an isoelectric point (PI) of 4.7–5.4, Sigma Aldrich, Saint Louis, MO, USA) referred to as S1, (b) a non-ionic polymer (57,000–66,000 g/mol, with a 98–99% purity, Alfa Aesar) termed S2, and (c) S3, an ionic polymer (300,000–400,000 g/mol, Alfa Aesar). All reagents were used as received, without any further purification, resulting in the Cu species summarized in Table 1.

#### 2.1.1. Synthesis of CuNPs Type 1, 3, and 4

The precursor salt was magnetically stirred for 15 min in deionized water to ensure complete dissolution. The pH of the aqueous stabilizer solution was adjusted to 10–11, using 0.5 M of sodium hydroxide. Then, the Cu solution was added dropwise to the stabilizer whilst stirring at ambient conditions and retaining the pH values between 9 and 11. This was sustained until the color of the solution changed to purple (for CuNP Type 1) and blue (for CuNPs Type 3 and 4), thus indicating the formation of Cu-NPs.

#### 2.1.2. Synthesis of CuNP Type 2

Similarly, copper salt and stabilizer were separately dissolved in deionized water but then rapidly added one to another. Then, the solution was magnetically stirred (at ambient conditions), and we adjusted its pH values to 10–11 through the controlled addition of 0.5 M of sodium hydroxide, resulting in the formation of CuNP Type 2 (colored bright green).

### 2.2. Physicochemical Characterization

Particle size, morphology, and shape were determined by High-Resolution Transmission Electron Microscopy (HR-TEM) on a JEOL JEM 2010 by Oxford INCA (Freising, Germany). Dynamic Light Scattering (DLS) was performed with VASCO 3 DLS analyzer by Cordouan Technologies (Pessac, France), providing information of the particle size distribution profiles. Attenuated Total Reflectance (ATR), performed on a Cary 630 FTIR Spectrometer by Agilent Technologies (Santa Cara, CA, USA) with a Diamond ATR sampling accessory, was employed to analyze the resulted copper-based nanoparticles. A Laser Doppler Electrophoresis (LDE) technique, using a Wallis Zeta analyzer by Cordouan (Pessac, France), was used to evaluate the surface potential of the resultant copper. According to the literature [33], zeta potential is a key indicator of electrostatic stabilization in colloidal systems.

### 2.3. Fungal Strains and Growth Conditions

Three strains of *F. oleagineum*, *C. gloeosporioides s.s*., and *C. acutatum s.s*. were used in the study. *F. oleagineum* strain was isolated from sporulating lesions on leaves of the local cultivar “Chalkidikis” and identified based on the morphological and cultural characteristics of the isolate as described by Graniti [34] and used in a previous study [32]. *C. gloesporioides s.s.*, and *C. acutatum s.s*. isolates were provided to us by Prof. Tsitsigiannis D., Agricultural University of Athens and were strains that had been isolated from mummified fruit of cv. “Kalamon” [35]. All the fungal strains were maintained on Potato Dextrose Agar (PDA) slants at 4 °C until use. For the inoculum production of *F. oleagineum*, the isolate was cultivated on Olive Leaf Extract (OLE) liquid substate for 10 days at 19 °C [36]. For inoculum production of *C. acutatum s.s*. and *C. gloeosporioides s.s*., the isolates were grown for 1 week on PDA at 23 °C with a 12 h photoperiod. For all the pathogens, inoculum was harvested in distilled sterile water.

### 2.4. In Vitro Fungitoxicity Tests

The effects of the four CuNPs listed in Table 1 on spore germination, germ tube growth, and mycelial growth were tested using the *C. gloeosporioides s.s*., *C. acutatum s.s*., and *F. oleagineum* isolates. Measurements of spore germination inhibition and germ tube length inhibition were performed on water agar (WA) 1.5%, while measurements of mycelial growth inhibition were conducted on PDA medium. As reference compounds, two commercial copper products were included in the study, copper oxide (Nordox 75 WG, K & N Efthimiadis, Thessaloniki, Greece) and copper hydroxide (Kocide 2000, 35 WG, K & N Efthimiadis, Thessaloniki, Greece). CuNPs stock solutions were added into autoclaved nutrient media to achieve concentrations of 10, 50, 100, and 200 μg·mL^−1^ for CuNPs Type 2 and Type 3, of 10, 50, 100, 200, and 400 μg·mL^−1^ for CuNP Type 4, and of 10, 50, 100, 200, 400, 600, and 800 μg·mL^−1^ for CuNP Type 1, by adding appropriate volumes of the fungicide stock solutions into the media while they were still liquid. Copper oxide and copper hydroxide were added into the nutrient media at concentrations of 0, 10, 20, 50, 100, 200, 400, 600, and 800 μg·mL^−1^ of Cu^++^. Control media were not amended with any product. In preliminary experiments, an additional set of nutrient media containing each of the 4 stabilizers, at doses of 10, 50, 100, and 200 μg·mL^−1^, were prepared to test for any inhibitory effect of the stabilizers on the mycelial growth, spore germination, and germ tube growth of the three pathogens.

To measure spore germination, spores were produced on PDA as previously described in Section 2.3. Spore suspensions for each isolate were prepared in sterile distilled water at a concentration of 1 × 10^5^ mL^−1^. Aliquots of the conidial suspensions were spread on product-amended and product-free 6 cm Petri dishes. After 24 h of incubation at 20 °C in the dark, conidia were checked for germination and germ tube growth. Conidia were considered as germinated when the germ tube length was at least half of the conidium length [24]. Measurements of germ tube length were conducted using a Carl Zeiss, AXIO Lab A1 microscope. One hundred conidia were counted per plate, and three replicate plates were prepared for each product concentration tested.

For the assessment of mycelial growth inhibition, mycelial plugs were removed, with the aid of a 5 mm diameter cork borer, from the colony margins of *F. oleagineum* and *Colletotrichum* spp., actively growing 25- and 7-day-old colonies, respectively, on PDA and placed upside down on the center of 9 cm dishes containing the fungicide-amended or -unamended media. Cultures of *F. oleagineum*, *Colletotrichum acutatum*, and *C. gloeosporioides* were incubated at 20 °C in the dark for 15 and 7 days, respectively. Then, the mean colony diameter was measured and expressed as a percentage of the mean diameter of the untreated control. Tests were replicated three times for each treatment.

### 2.5. In Vivo Effect on Fusicladium oleagineum

One-year-old olive trees (cv. Chalkidikis) were used to evaluate the protectant activity of CuNPs against *F. oleagineum*. The trees were produced by a commercial olive nursery company and planted in 3 L plastic pots. Trees were sprayed with a hand sprayer to run-off with CuNPS and reference products. The application dose for all Cu formulation products was 240 μg·mL^−1^ active ingredient (a.i.). The applied concentration was common for all the treatments and was selected based on the maximum applicable concentration that could be reached for CuNPs. Control plants were sprayed with distilled water. All the plants were artificially inoculated 24 h after the fungicide application by spraying them with a conidial suspension (2 × 10^6^ spores/mL) amended with Tween 80 (0.2 μL mL^−1^) as surfactant, using a fine hand sprayer. For each treatment, 5 replicate plants were used, and the experiment was repeated twice.

After artificial inoculation, the plants were covered with plastic bags and incubated in a plant growth chamber at 23 ± 2 °C and 100% RH for 48 h in the dark. Then, the bags were removed, and the plants remained in the growth chamber at 19 °C, 70% RH, and 14/10 L:D photoperiod for 3 months, following a protocol described previously [15]. Evaluation of the efficacy of CuNPs and Cu reference products against the olive leaf spot was conducted by measuring the number of leaves with latent infections of pathogen. Latent infections were determined using the NaOH method [37]. From each plant, the 15 younger fully expanded leaves that had been treated and inoculated were removed from the plants and transferred for 30 min in a 5% NaOH solution. By this treatment, olive leaf spot lesions became evident, and the number of leave showing disease symptoms were counted to determine disease incidence (%).

### 2.6. In Vivo Effect on Colletotrichum acutatum s.s.

The effect of CuNPs on the control of anthracnose caused by *C. acutatum s.s.* was measured in an experiment conducted in a 20-year-old olive orchard (cv. Chalkidikis) located in the region of Thessaloniki. Experimental plots were consisting of three replicate trees per treatment and five branches per tree. There were three replicate plots per treatment. During the experimentation period (April to July 2020), no fungicides were applied on the trees.

Branches were sprayed to run-off with CuNPs and reference Cu products at full bloom (BBCH 65) stage [38]. As in the case of *F. oleagineum*, the application dose for all the products included in the study was 240 μg·mL^−1^ active ingredient (a.i.). Artificial inoculations were conducted on the inflorescences 24 h after the product applications, spraying a conidial suspension (2 × 10^6^ spores/mL) amended with Tween 80 (0.2 μL mL^−1^) as surfactant. The branches bearing the inoculated inflorescences were covered with thin polythene bags to provide moist conditions for 12 h. The efficacy of the applied products in controlling anthracnose was assessed during two distinct developmental stages. The first assessment was conducted seven days after the artificial inoculation on the inflorescences and the second assessment was conducted on young fruit (July 2020). Disease incidence on the inflorescences was measured following a procedure described previously [39]. Briefly, 10 inflorescences per branch (150 inflorescences per treatment) were removed from the branches and incubated in closed plastic containers (100% RH) at 23 °C for 5 days. At the end of the incubation period, the infected inflorescences were scored by measuring the number of inflorescences with grayish green mycelial growth. The second assessment was conducted by measuring fruit showing latent infections following a procedure described previously by Moral et al. [23]. Fruit (10 fruits per branch) were collected at BBCH75 (July 2020) when they had approximately 50% of the final size [38]. Then, fruit were transferred to the laboratory and immersed in a solution containing the herbicide 1,1′-ethylene-2,2′-bipyridyldiylium dibromide (Diquat) for 1 min. After treatment, the fruit were incubated in a humid chamber (100% RH) at 23 °C in the dark for 21 days. At the end of the incubation period, the number of fruit showing anthracnose symptoms (rotten tissues covered by conidia in a gelatinous matrix or covered by abundant white-gray mycelium on the fruit surface) were measured. Disease incidence of fruit with latent infections was calculated as the percentage of fruit that showed anthracnose symptoms with respect to the total number of treated fruits.

### 2.7. Data Analysis

In the in vitro sensitivity measurement experiments, effective concentrations causing 50% inhibition (EC_50_ values) of mycelial growth, spore germination, and germ tube length for each isolate and fungicide were calculated by plotting the relative inhibition of either mycelial growth, spore germination, or germ tube length against the Log10 fungicide concentrations in 3 independent measurements. Calculations of EC_50_ values were performed using SAS (JMP, SAS Institute, Cary, NC, USA). The EC_50_ values were subjected in an analysis of variance (ANOVA), and means were compared by Fisher’s Least Significant Difference (LSD) test carried out on log-transformed EC_50_ values and then converted to original scale values. Percentage control values of in planta and field experiments were calculated as 100—incidence (treated)/incidence (untreated). Percentage data were arcsine-transformed for statistical analysis. Data of each replication were combined after testing for homogeneity of variance using Levene’s test. Fisher’s LSD test was used to determine significant differences between treatments at *p* = 0.05. All the statistical analysis tests were performed using SPSS Statistics version 11.0 (IBM, New York, NY, USA).

## 3. Results

### 3.1. Dynamic Light Scattering

**Figure 1:**
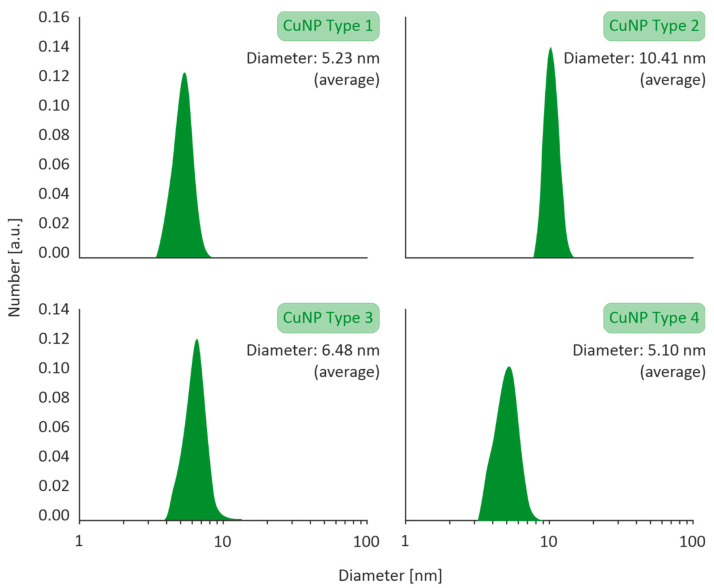
Particle size distribution of Type 1–4 CuNPs assessed by Dynamic Light Scattering (DLS) with a VASCO 3 analyzer by Cordouan Technologies.

The size distribution profiles of the CuNPs are illustrated in Figure 1. All types of CuNPs exhibited closely clustered population of monodispersed NPs, with average diameters ranging in between 5.10 and 10.41 nm.

### 3.2. Transmission Electron Microscopy

**Figure 2:**
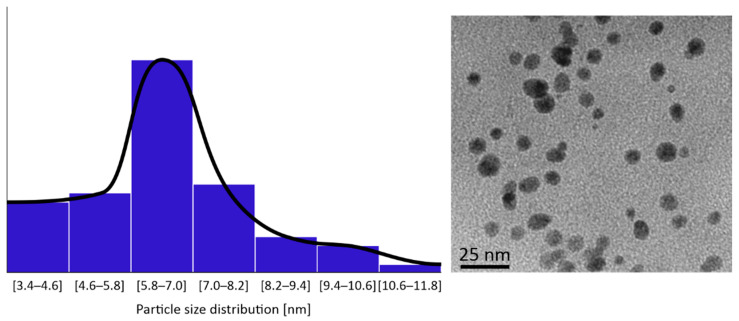
Indicative CuNPs shapes and size distributions as observed by HR-TEM on a JEOL JEM 2010 by Oxford INCA.

HR-TEM images of the CuNPs indicated that all the four types were of a spherical morphology, with an average size between 5 and 10 nm. An HR-TEM image of CuNPs along with its image-based size distribution is indicatively depicted in Figure 2. It should be noted that TEM measurements are expected to lead to slightly smaller size distributions of the NPs than DLS (shown in Figure 1), as DLS measures a hydrodynamic diameter of the NP.

### 3.3. AΤR Measurements

The ATR method was utilized to detect the characteristic peaks of CuNPs. ATR spectra of CuNPs Types 1 and 2 presented similar stretching and bending frequencies to a previous study within our group [5], showing, in brief, the presence of four characteristic bands of stabilizer S1 as well as the symmetrical stretching and bending vibration of Cu-O bond (CuNP Type 1). In the same context, ATR spectrum of CuNP Type 2 indicated that copper oxide and copper hydroxide are the prevalent copper species and presented the six characteristic peaks of stabilizer S2.

**Figure 3:**
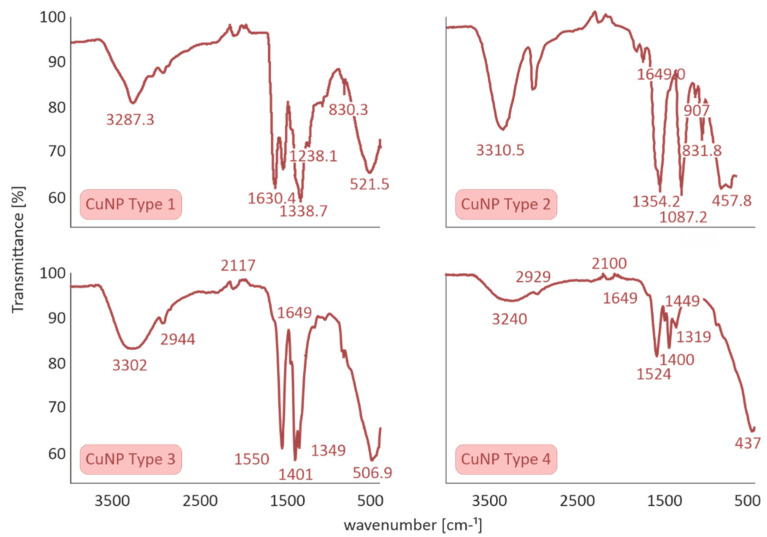
Attenuated Total Reflection (ATR) spectra of all four CuNPs species performed on a Cary 630 FTIR Spectrometer by Agilent Technologies.

With respect to the above, the ATR spectra of CuNPs Types 3 and 4 were evaluated to determine the interaction of S3 with CuNPs. The ATR spectra of all four Cu species are summarized in Figure 3.

A sharp band at ≈1400 cm^−1^ can be attributed to carbonyl stretching, which is assigned to stabilizer S3. According to the literature, pure S3 presents two sharp characteristic bands at 1600–1700 cm^−1^ assigned to symmetric and asymmetric stretching of COO groups, while the presence of a shoulder peak at 1649 cm^−1^ and a significant shifting at ≈1550 cm^−1^ is clearly illustrated. At the molecular level, these significant changes could be attributed to the efficient incorporation of stabilizer S3 to CuNPs Types 3 and 4.

### 3.4. Laser Doppler Electrophoresis

The surface charge of CuNPs was estimated by Laser Doppler Electrophoresis technique (LDE) in deionized water. As depicted in Table 1, all four samples presented a negative surface charge from −27.09 to −1.47 mV. Even though CuNPs of Types 1 and 2 possess low zeta potential values, these systems present high stability through time due to steric forces induced by stabilizers S1 and S2, respectively. On the other hand, the highly negative surface charge of CuNPs of Types 3 and 4 could be attributed to the electrostatic stabilization of nanoparticles by S3, which is an anionic polymer of high molecular weight. The physicochemical characteristics of all four synthesized CuNPs are presented at Table 1.

### 3.5. Short-Term Stability of CuNPs in Farming/Tap Water

**Figure 4:**
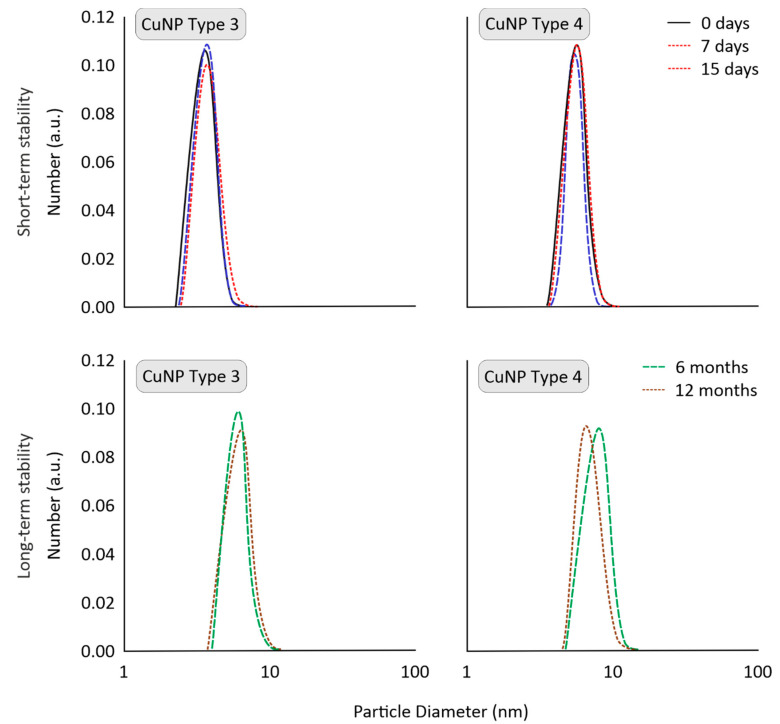
Short-term stability (in tap water) and long-term stability (upon storage), indicatively for CuNPs Types 3 and 4, evaluated by Dynamic Light Scattering (DLS).

In the present study, the short-term stability of CuNPs was evaluated after their two-fold dilution in tap water. CuNPs stability was estimated at 25 °C for 15 days to observe any aggregation (visual observation) or extreme changes in average size and size distribution. CuNPs Types 1, 3, and 4 remained stable with respect to optical observation (no changes in color or aggregation), size, and size distribution (see Figure 4). This important aspect could be attributed to the synergistic effect of stabilizer on both the surface of CuNPs (small size and stable nanoparticle) and also on the final formulation (homogeneous stable dispersion). Only CuNPs Type 2 were mildly affected as to their size distribution profiles. The size distribution of CuNPs Types 3 and 4 are indicatively presented in Figure 4.

### 3.6. Storage Stability of CuNPs

The long-term physical stability of CuNPs is critical to evaluate the scalability potential of their production route, which is vital for their commercialization. The storage stability of CuNPs was monitored for 1 year (at 25 °C). To resemble realistic conditions, stability was examined for high concentrations, as pesticides are typically stored as solids or highly concentrated. No significant changes were catalogued in CuNPs Types 1, 3, and 4, which can be attributed to the use of stabilizers during their synthesis and storage. As depicted in Figure 4, CuNPs presented stable size distribution profiles (determined via DLS). Τhis would suggest that they could be commercialized as an alternative to conventional pesticides.

### 3.7. In Vitro Effects of CuNPs on Mycelial Growth, Spore Germination, and Germ Tube Length

**Figure 5:**
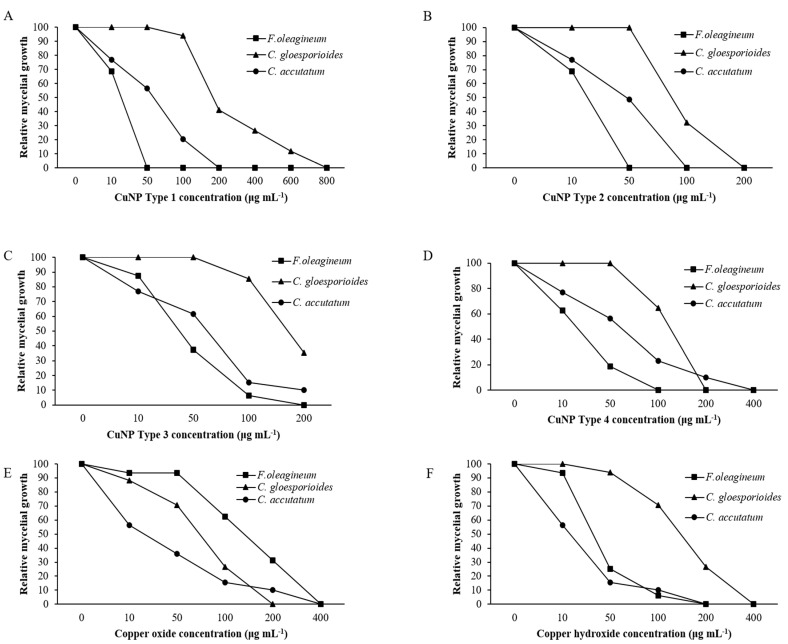
Mean relative mycelial growth of *Fusicladium oleagineum*, *Colletotrichum acutatum s.s.*, and *Colletotrichum gloesporioides s.s.* isolates on artificial nutrient media amended with several concentrations of CuNP Type 1 (**A**), CuNP Type 2 (**B**), CuNP Type 3 (**C**), CuNP Type 4 (**D**), Copper oxide (**E**), and Copper hydroxide (**F**).

**Figure 6:**
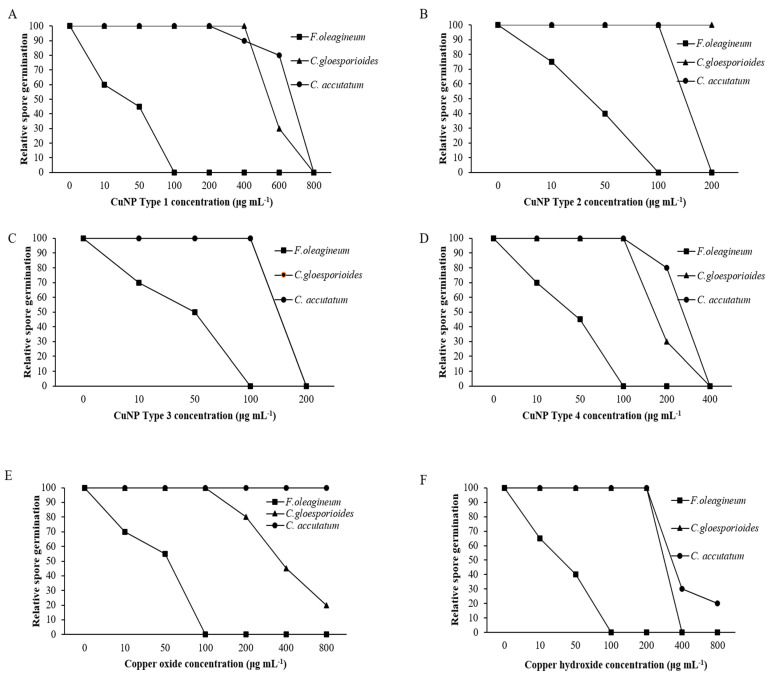
Mean relative spore germination of *Fusicladium oleagineum*, *Colletotrichum acutatum s.s.,* and *Colletotrichum gloesporioides s.s.* isolates on artificial nutrient media amended with several concentrations of CuNP Type 1 (**A**), CuNP Type 2 (**B**), CuNP Type 3 (**C**), CuNP Type 4 (**D**), Copper oxide (**E**), and Copper hydroxide (**F**).

**Figure 7:**
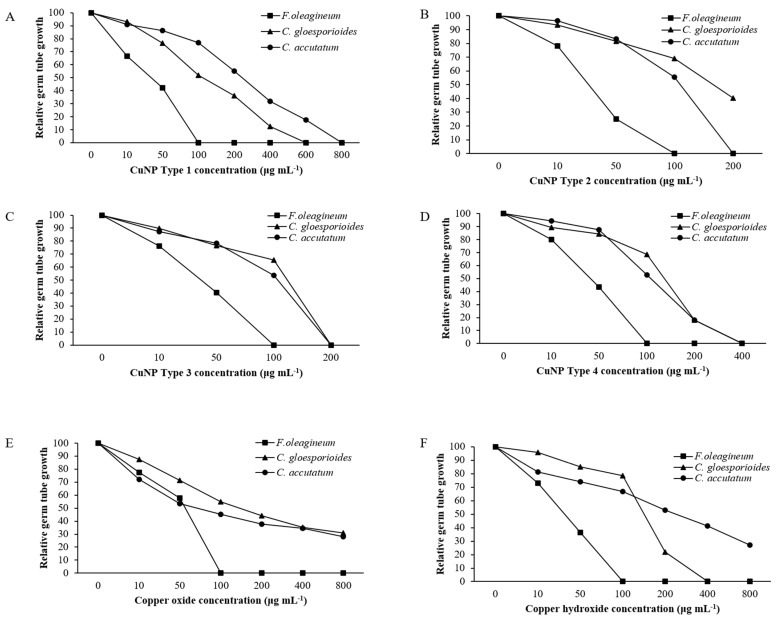
Mean relative germ tube growth of *Fusicladium oleagineum*, *Colletotrichum acutatum s.s.,* and *Colletotrichum gloesporioides s.s.* isolates on artificial nutrient media amended with several concentrations of CuNP Type 1 (**A**), CuNP Type 2 (**B**), CuNP Type 3 (**C**), CuNP Type 4 (**D**), Copper oxide (**E**), and Copper hydroxide (**F**).

Mycelial growth was the developmental stage most severely affected by exposure of *F. oleagineum* to CuNPs (Figure 5). Among the CuNPs tested, Types 1 and 2 were the most active, since they completely inhibited the mycelial growth of *F. oleagineum* at 50 μg·mL^−1^ (Figure 5A,B), with mean EC_50_ values of 25.9 and 25.8 μg·mL^−1^, respectively (Table 2). For the remaining CuNPs tested, complete inhibition of mycelial growth was observed in quite higher concentrations ranging from 100 to 200 μg·mL^−1^, while the two commercial products were the least effective in mycelial growth inhibition (Figure 5C–F). Spore germination and germ tube elongation in *F. oleagineum* were less severely affected by the tested CuNPs. For all the compounds tested, including the reference compounds of copper oxide and copper hydroxide, spore germination and germ tube elongation were completely inhibited by the concentration of 100 μg·mL^−1^ (Figure 6 and Figure 7). The four tested stabilizers did not affect any developmental stage tested of neither *F. oleagineum* nor any of the two *Colletotrichum* spp., since in all the doses tested, the fungal responses were similar to that on water-containing control media (data non shown).

In *C. acutatum s.s*., mycelial growth was by far the developmental stage most severely affected by the tested CuNPs. EC50 values of the CuNPs tested against mycelial growth ranged from 50 to 75.3 μg·mL^−1^, while the respective values against germ tube growth and spore germination ranged from 102 to 225 and 150 to 725 μg·mL^−1^ (Table 2). CuNP Type 2 at the concentration of 100 μg·mL^−1^ completely inhibited the mycelial growth of the *C. acutatum s.s.* isolate (Figure 5B), while the remaining compounds completely inhibited the mycelial growth at the concentration of 200 μg·mL^−1^ (Figure 5A,C,F) or even higher than 200 μg·mL^−1^ (Figure 5D,E). Higher concentrations were required to achieve complete inhibition of spore germination or germ tube elongation by all the compounds tested. However, even at these developmental stages, some of the CuNPs tested were more active than the reference compounds. For instance, the complete inhibition of spore germination and germ tube elongation was achieved by 200 μg·mL^−1^ of CuNP Type 2 (Figure 6B and Figure 7B) or CuNP Type 3 (Figure 6C and Figure 7C), while for the reference compounds, copper oxide and copper hydroxide minimum inhibitory concentrations of either spore germination or germ tube elongation were higher than 800 μg·mL^−1^ (Figure 6E,F and Figure 7E,F).

Measurements of mycelial growth, spore germination, and germ tube elongation inhibition for the *C. gloesporioides* isolate exposed to the fungicide concentrations tested showed that this fungal species was the least sensitive to CuNPs tested. Measurements of EC_50_ based on the inhibition of mycelial growth, spore germination, and germ tube growth revealed values higher than 100 μg·mL^−1^ for all the tested products and developmental stages except CuNP Type 2 and copper oxide, for which EC_50_ measurements based on the inhibition of mycelial growth revealed values of 75.6 and 72.5 μg·mL^−1^, respectively (Table 2). No marked differences were observed in the MIC values that ranged from 200 to >800 μg·mL^−1^ for the three developmental stages tested (Figure 5, Figure 6 and Figure 7).

### 3.8. In Vivo Effect on F. oleagineum

**Figure 8:**
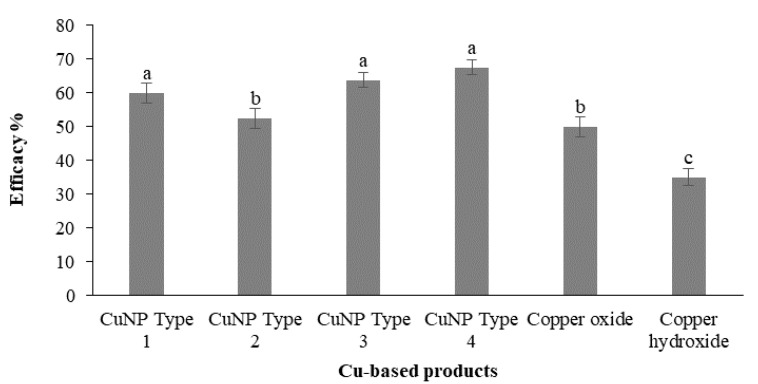
Efficacy (%) of Cu-NPs and conventional Cu-product treatments against *Fusicladium oleagineum* in experiments conducted in one-year-old olive trees (cv. Chalkidikis) incubated in a plant growth chamber. Treatments’ efficacy (%) assessments were based on the disease incidence measurements on the untreated control plants. Different letters on the columns indicate significant differences according to Fisher’s LSD test at *p* = 0.05. Vertical lines indicate the standard error of the mean.

Artificial inoculations with the *F. oleagineum* strain used in the experiment were successful. Disease symptoms appear on control plants almost 2 months after the inoculation. As expected, the higher disease incidence values were observed in the untreated control plants. All the chemical treatments either using CuNPs or the reference conventional copper fungicides reduced disease incidence compared to the untreated control. The higher (*p* < 0.05) control efficacy values were achieved by the application of CuNP Type 4, CuNP Type 3, and CuNP Type 1, with control efficacy values ranging from 60 to 67.5% (Figure 8). CuNP Type 2 provided a control efficacy that was similar (*p* > 0.05) to that of the conventional copper oxide compound used as a reference product, while the lowest control efficacy was achieved by the conventional copper hydroxide product with a control efficacy value of only 35% (Figure 8).

### 3.9. In Vivo Effect on Colletotrichum spp.

**Figure 9:**
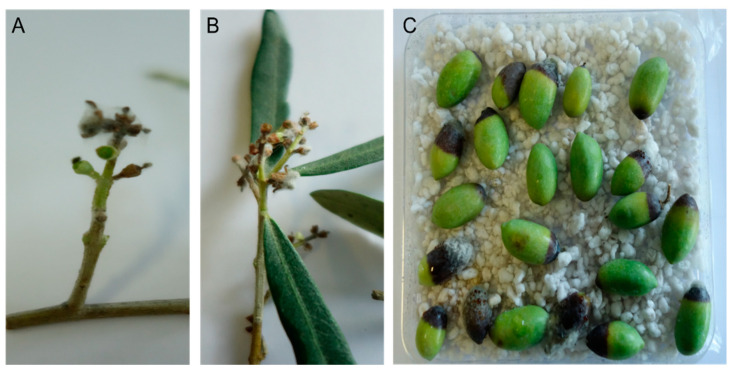
(**A**,**B**) Symptoms of anthracnose on olive blossoms 7 days after the artificial inoculation with *Colletotrichum acutatum* and (**C**) on young olive fruit exposed to diquat treatments.

**Figure 10:**
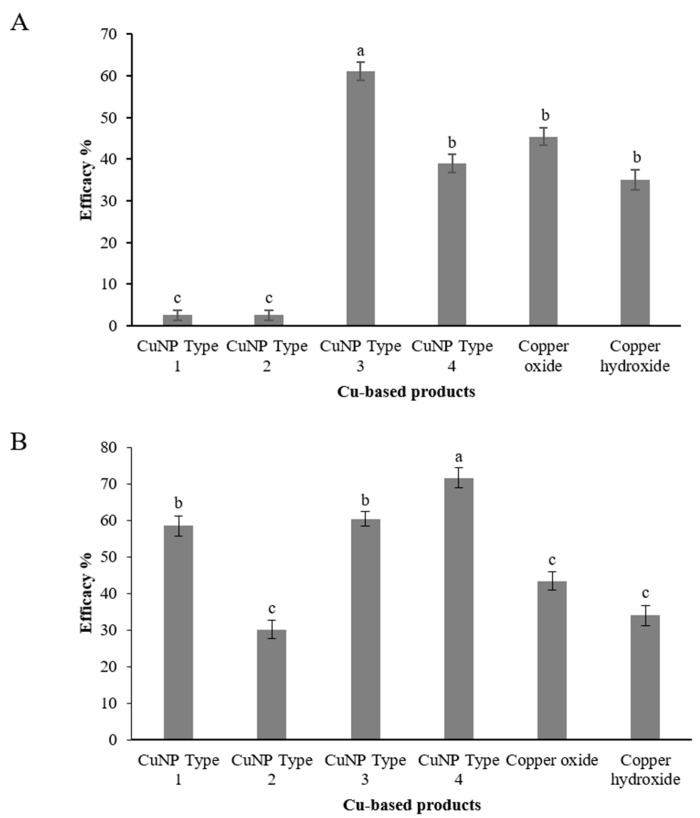
Efficacy (%) of Cu-NPs and conventional Cu-product treatments against *Colletotrichum accutatum* *s.s.* on (**A**) olive inflorescences and (**B**) on olive fruit. Treatments’ efficacy (%) assessments were based on the disease incidence measurements on the untreated control plants. Different letters on the columns indicate significant differences according to Fisher’s LSD test at *p* = 0.05. Vertical lines indicate the standard error of the mean.

Assessment of CuNPs performance against *C. acutatum* was conducted in two distinct phenological stages of olive tree. Taking into account recent epidemiological data on the role of blossom infections for the onset of the disease, artificial inoculations were conducted at the blossom stage, and disease was assessed both on blossoms and on fruit as latent infections (Figure 9). Artificial inoculation on the blossoms resulted in a high disease incidence on the untreated control trees. The CuNP Type 3 was found to be the most effective in protecting blossoms from anthracnose attacks with a control efficacy value of 61.04% (Figure 10A). A second CuNP (Type 4) provided a control efficacy similar to that achieved by the reference treatments of copper oxide and copper hydroxide, while the remaining CuNPs were ineffective in reducing blossom necrosis symptoms imposed by artificial inoculations with C. acutatum (Figure 10A). Further evidence for the success of artificial inoculations on blossoms and the control efficacy provided by some of the treatments was the fact that great visible differences were observed in fruit setting among the several treatments (data not shown).

Assessment of latent infections on olive fruit inoculated with *C. acutatum* and treated with CuNPs and reference products resulted in a greater variability. CuNP Type 4 was the product ensuring the higher control efficacy against anthracnose with a control efficacy value of 71.7% (Figure 10B). Two more CuNPs, CuNP Type 1 and CuNP Type 3, provided control efficacy of 58.5 and 60.4%, respectively. The lower control efficacy values were provided by the two reference products, copper oxide and copper hydroxide, and the CuNP Type 2 with values ranging from 30.2 to 43.4% (Figure 10B).

## 4. Discussion

Olive crop is frequently treated with copper fungicides, since quite a high number of copper spray applications is required during spring and autumn periods to successfully control major foliar or fruit diseases such as anthracnose or peacock spot. However, this leads to an increased risk for the accumulation of high copper concentrations in the olive orchards’ environment and in particular olive groves soil [7]. Despite the widely accepted need for a reduction of Cu accumulation in the olive groves environment, research related to the development of methods or means that could enable the achievement of this target is limited [25,40]. Taking into account that the reduction of soil contamination by heavy metals is a priority, in the current study, the development of some novel CuNPS was accomplished, and they were tested for their efficacy against anthracnose and peacock spot of olive.

All types of CuNPs presented prolonged storage stability except for CuNP Type 2, which was slightly affected for size distribution measurements by DLS. Despite their small size, no aggregation or optical changes were observed, indicating the importance of a steric and/or electrostatic stabilization of nanoparticles by stabilizers.

In the present study, we also evaluated one of the most challenging aspects of using nanoparticles in agriculture, meaning their stability after dilution in tap water. Most pesticides are solid or concentrated formulations and need to be dispersed or diluted in water before application (e.g., sprayable form). In general, deionized or distilled water is used to synthesize nanoparticles to avoid undesirable interactions with prevalent ions and impurities of tap water, which may result in aggregates during preparation. However, the end users in agricultural applications (e.g., farmers) dilute bulk material with tap or well water, as purer water types require chemical/physical treatments, creating an additional burden with respect to complexity and cost-efficiency. Thus, in the framework of this research, stable CuNPs were developed concerning their short-term stability after dilution in tap water, indicating once again the significance of using stabilizing molecules in nanoparticle synthesis.

The synthesized CuNPs presented a mean diameter range between 4.90 and 10.41 nm. The narrow size distribution and small size ensure a higher antimicrobial activity [5]. The resulted CuNPs were characterized among the lowest diameters reported in the literature [12,41,42,43]. In addition, previous studies have shown that size is a key factor for their antimicrobial activity, since the smaller the size, the easiest their penetration into cells and the higher their activity [4,44].

In vitro effects of the tested CuNPs on the mycelial growth, spore germination, and germ tube elongation provided the first line of evidence related to their potential toxicity and efficacy against the three olive pathogens tested. The measurement of mycelial growth inhibition or spore germination assays on media containing increasing concentrations of the tested nanoproducts is an efficient method of antifungal activity assessments and has been used in numerous previous studies aiming to determine the antifungal activity of several nanoparticles [3,4,45]. Our results supported that most of the developed CuNPs showed a higher activity compared to that of commercially available conventional copper products. The observed antifungal activity of these products was exhibited in a dose-dependent manner, which is in agreement with findings of other studies reporting on the antifungal activity of metallic nanoparticles against fungal species such as *Thielaviopsis basicola*, *Phytopththora nicotianae* or *Fusarium graminearum* [4,46]. Interestingly, the mycelial growth stage was found to be more sensitive to the action of CuNPs compared to spore germination and to a lesser extent to germ tube growth. This absence of Cu fungicides activity against spore germination or germ tube elongation is in agreement with findings of Obanor et al. [16], suggesting that copper hydroxide and copper sulfate were ineffective in inhibiting spore germination in *F. oleagineum*. The strong activity of CuNPs against fungal mycelia have been observed in several fungal species such as *Fusarium oxysporum*, *F. solani*, and *Neofusicoccum* spp. [11], while similarly, silver nanoparticles had also showed a stronger activity against mycelial growth compared to that against spore germination in *F. graminearum* [45]. In the study of Pariona et al. [11], the exposure of fungal mycelial to low concentrations of CuNPs caused a light deformation of the mycelium surface by the formation of unusual bulges, while at higher concentrations, a strong deformation of the mycelium was observed that promoted the outflow of the intercellular components and the shrinkage of the mycelial hyphae. The mechanism of CuNPs and microbe interactions has not been precisely established yet [8]. However, from several studies, a strong line of evidence has been obtained associating toxic effects on microbial cells with increased ROS generation by CuNPs [11,14,47]. Sensitivity comparisons among the three pathogens tested in vitro showed that *F. oleagineum* was the most sensitive to the copper compounds tested, followed by *C. acutatum*, while *C. gloesporioides* was the least sensitive. This finding is in agreement with previous reports comparing the intrinsic activity of copper compounds against major olive pathogens [25,26]. The observed variability of CuNPs efficacy in controlling the growth of the three fungal species tested is not surprising. Similar differences in the efficacy of metal nanoparticles have been observed against several fungal species tested previously [3,11,48]. The differences in the inhibitory effect of the CuNPs against the different fungal species tested may be explained by differences in the fungal cell walls that lead to differences in the behavior toward oxidative stress imposed by the CuNPs, as has been proposed by Pariona et al. [11]. However, further research is required to elucidate the precise mechanism of action of CuNPs against the tested pathogens and possible implications of fungal cell wall architecture and composition with the observed inhibitory effects.

The results of the in planta trials against olive leaf spot and anthracnose diseases strongly suggest that at least two of the evaluated products (CuNP Type 3 and CuNP Type 4) exhibited a performance superior to that of conventional copper fungicides. The observed superior performance of CuNP Type 3 or Type 4 was achieved with applications at a rate of 240 μg·mL^−1^, which was significantly lower than the commercially recommended rates of 1120 μg·mL^−1^ for copper oxide or the rate of 1190 μg·mL^−1^ for copper hydroxide. Thus, they could replace them as new and innovative antimicrobial agents. During the last decade, numerous CuNPs have been evaluated against fungal plant pathogens; however, most of these reports were related to the in vitro antimicrobial effect of CuNPs [3,49]. In contrast, related limited is the information on the in planta effects of CuNPs against fungal or Oomycete pathogens such as *Phytophthora infestans*, *Verticillium dahliae*, *Botrytis cinerea*, or *Podosphaera panosa* [9,50,51,52]. Furthermore, it is of particular importance that the efficacy of the products at least against *C. acutatum* was tested under field conditions.

In the current study, only the protectant activity of CuNPs was tested against the two important olive pathogens by applying them 24 h before the artificial inoculation of the plants. However, further studies are required to determine whether some curative action is also exhibited by these CuNPs. Copper compounds are mainly considered as protectant fungicides. However, in some cases, a curative activity was observed, in particular, when copper compounds were applied in mixture with fungicides of other chemical classes [53]. The tested pathogens and in particular *F. oleagineum* are of relatively low growth rate, and therefore, curative applications that are conducted 1–2 days after inoculation may provide a satisfactory control efficacy.

The use of nano-based material for agricultural purposes is currently restricted because of the absence of comprehensive and detailed knowledge of the interactions between nanomaterials and living organisms [1]. For instance, different cases of toxic effects have been reported on several plants treated with metal nanoparticles [54,55], while little is known on the effects of nanoparticles on human or animal health. Several factors such as the concentration, size, or the shape of the nanoparticles shape the risk for the toxicity of them [8]. In our study, none of the tested CuNPs showed any toxic effect on olive plants at the applied concentrations. However, more detailed studies with more application concentrations are required to obtain full evidence on the absence of toxic effects.

## 5. Conclusions

In conclusion, in the current study, four CuNP types were developed through a slightly modified wet chemistry method. This technique offers significant advantages over other methods, such as simplicity, flexibility, and ease of transition to a higher production scale. The CuNPs were characterized in reference to Transmission Electron Microscopy, Dynamic Light Scattering, Laser Doppler Electrophoresis, and Attenuated Total Reflection measurements. The physicochemical characterization of the CuNPs presented a spherical morphology with a size range between 4.9 and 10.41 nm, depending on the CuNP type. Two of these products (CuNP Type 3 and CuNP Type 4) were found to be active against both peacock spot and anthracnose both in vitro and in planta. The control efficacy that they provided was higher than that of commercial copper products. Thus, the developed CuNPs could reshape disease control in olive culture by replacing traditional copper fungicides with the nano-enabled counterparts, and in this way, they could contribute to the reduction of copper accumulation in the environment. However, further research is required to determine the environmental profile of these products along with field experiments data that will determine their efficacy and safety for the environment. Similarly, further research is required to determine the precise mechanism of antifungal activity of CuNPs and how the physicochemical characteristics of the CuNPs affect their performance against these major olive pathogens.

## Figures and Tables

**Table 1:** Cu-NP species/types based on the employed synthesis route and their physicochemical characteristics.

CuNP Name	Precursor Salt	Stabilizer	Concentration (ppm)	Physicochemical Characteristics		
				pH	Size (nm)	Zeta Potential (mV)
Type 1	Copper Nitrate (CN)	S1 ^1^	300	10.0–10.5	5.23 ± 0.8	−12.23 ± 0.9
Type 2	Copper Nitrate (CN)	S2 ^2^	300	10.0–10.5	10.41 ± 1.2	−4.64 ± 0.4
Type 3	Copper Nitrate (CN)	S3 ^3^	300	10.0–10.5	4.90 ± 0.2	−16.44 ± 0.8
Type 4	Copper Chloride (CC)	S3 ^3^	600	10.0–10.5	6.61 ± 0.6	−27.09 ± 0.9

^1^ S1 being an animal protein; ^2^ S2 being a non-ionic polymer and ^3^ S3 being an ionic polymer.

**Table 2:** Effective concentrations (μg·mL^−1^) of Cu^++^ and Cu-based nanoparticle (CuNP) fungicides, causing 50% inhibition (EC_50_ values) of mycelial growth, spore germination, and germ tube growth of *Fusicladium oleagineum*, *Colletotrichum acutatum s.s.*, and *C. gloeosporioides s.s.* isolates.

Cu-Based Products	Pathogen								
	*Fusicladium oleagineum*			*Colletotrichum acutatum s.s.*			*Colletotrichum gloeosporioides s.s.*		
	Mycelial Growth	Germ Tube Growth	Spore Germination	Mycelial Growth	Germ Tube Growth	Spore Germination	Mycelial Growth	Germ Tube Growth	Spore Germination
CuNP Type 1	25.9 a *	32.5 ab	42.5 ab	75.3 c	225 b	725 a	185.6 b	100 a	520 c
CuNP Type 2	25.8 a	22.8 a	35 a	50 ab	112 a	150 a	75.6 a	154 b	>200
CuNP Type 3	30 a	35.5 ab	50 b	70.6 c	109 a	150 a	180.9 b	135 ab	150 a
CuNP Type 4	45.5 a	45 b	43.5 ab	65.8 bc	102 a	321 b	150 b	150 b	110.5 a
Copper oxide	182.6 b	74.3 c	45 b	25.5 a	92 a	>800	72.5 a	124 ab	382.5 b
Copper hydroxide	30.5 a	36.7 ab	40.5 ab	25.5 a	265 b	345.5 b	135.9 ab	167 b	300 b

* Mean EC_50_ values followed by different letter in the column are significantly different according to Fisher’s LSD test at *p* = 0.05.

## Data Availability

The data presented in this study are available in this article.
